# Divergent Age-Dependent Conformational Rearrangement within Aβ Amyloid Deposits in APP23, APPPS1, and *App*^*NL-F*^ Mice

**DOI:** 10.1021/acschemneuro.4c00104

**Published:** 2024-04-23

**Authors:** Farjana Parvin, Samuel Haglund, Bettina Wegenast-Braun, Mathias Jucker, Takashi Saito, Takaomi C. Saido, K. Peter R. Nilsson, Per Nilsson, Sofie Nyström, Per Hammarström

**Affiliations:** † Department of Physics, Chemistry and Biology (IFM), Linköping University, 58183 Linköping, Sweden; ‡ German Center for Neurodegenerative Diseases (DZNE), University of Tübingen, 72076 Tübingen, Germany; § Hertie Institute for Clinical Brain Research, University of Tübingen, 72076 Tübingen, Germany; ∥ Laboratory for Proteolytic Neuroscience, RIKEN Center for Brain Science, Wako 351-0198, Saitama, Japan; ⊥ Department of Neurocognitive Science, Nagoya City University Graduate School of Medical Sciences, Nagoya 467-8601, Aichi, Japan; # Department of Neurobiology, Care Sciences and Society, Division of Neurogeriatrics, Karolinska Institutet, 17177 Solna, Sweden

**Keywords:** Alzheimer’s Disease, Aβ amyloid polymorphism, mouse models, plaque morphology, fluorescence imaging

## Abstract

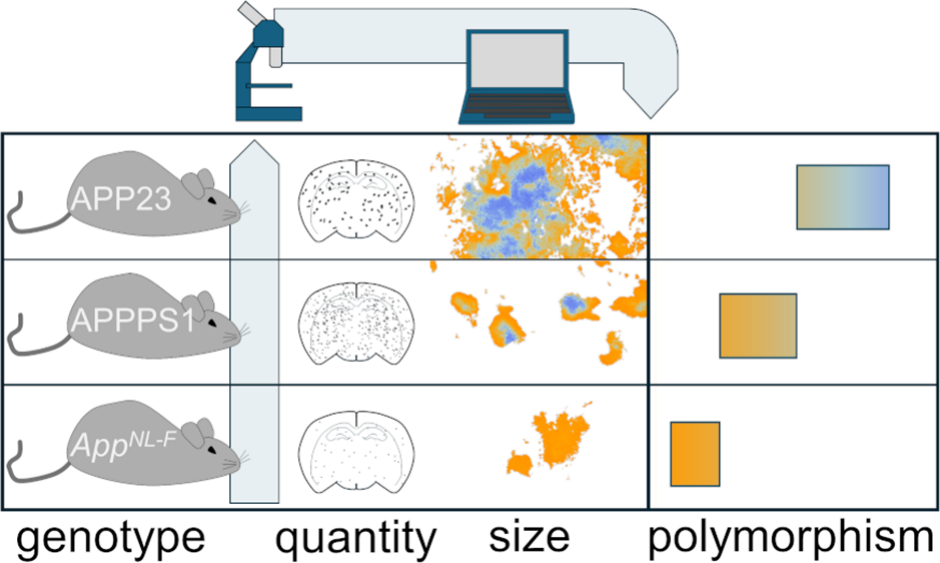

Amyloid plaques composed of fibrils of misfolded Aβ peptides are pathological hallmarks of Alzheimer’s disease (AD). Aβ fibrils are polymorphic in their tertiary and quaternary molecular structures. This structural polymorphism may carry different pathologic potencies and can putatively contribute to clinical phenotypes of AD. Therefore, mapping of structural polymorphism of Aβ fibrils and structural evolution over time is valuable to understanding disease mechanisms. Here, we investigated how Aβ fibril structures in situ differ in Aβ plaque of different mouse models expressing familial mutations in the AβPP gene. We imaged frozen brains with a combination of conformation-sensitive luminescent conjugated oligothiophene (LCO) ligands and Aβ-specific antibodies. LCO fluorescence mapping revealed that mouse models APP23, APPPS1, and *App*^*NL-F*^ have different fibril structures within Aβ-amyloid plaques depending on the AβPP-processing genotype. Co-staining with Aβ-specific antibodies showed that individual plaques from APP23 mice expressing AβPP Swedish mutation have two distinct fibril polymorph regions of core and corona. The plaque core is predominantly composed of compact Aβ40 fibrils, and the corona region is dominated by diffusely packed Aβ40 fibrils. Conversely, the AβPP knock-in mouse *App*^*NL-F*^, expressing the AβPP Iberian mutation along with Swedish mutation has tiny, cored plaques consisting mainly of compact Aβ42 fibrils, vastly different from APP23 even at elevated age up to 21 months. Age-dependent polymorph rearrangement of plaque cores observed for APP23 and APPPS1 mice >12 months, appears strongly promoted by Aβ40 and was hence minuscule in *App*^*NL-F*^. These structural studies of amyloid plaques in situ can map disease-relevant fibril polymorph distributions to guide the design of diagnostic and therapeutic molecules.

## Introduction

Alzheimer’s disease (AD) is a progressive neurodegenerative disease that affects millions of people worldwide. The manifestation of AD is complex, and clinical signs span across cognitive, personality, and behavioral changes, and motoric disturbances. Biochemical alterations, pathophysiological hallmarks, and neuroinflammation are obvious.^1,2^ The major histopathologic findings in AD brain are Aβ-amyloid plaques (hereafter termed plaques), neurofibrillary tau tangles (hereafter termed tangles), and often cerebral amyloid angiopathy (CAA) from Aβ fibrils in vasculature. The formation of Aβ plaques is tightly linked to the disease.^3^ However, this pathological hallmark of AD is also commonly found in healthy elderly,^4^ justifying the question of how plaque structures differ between healthy and diseased individuals and why.^5^ Although the link between plaque pathology and AD was first described by Alois Alzheimer in 1906 it is only during the later decades that a plethora of plaque morphotypes has been described systematically.^6^ Aβ plaque and CAA microscopic morphology is likely associated with amyloid fibril structural polymorphism which is widespread for Aβ fibrils formed in vitro^7^ and in vivo.^8^

Conformation-sensitive amyloid ligands, luminescent conjugated oligothiophenes (LCOs), entail several benefits over conventional methods for staining ex vivo amyloids in situ. When bound to amyloids, these molecules are highly photostable. The flexible molecular backbone of LCOs allows tight binding to amyloid fibrils, which render variable fluorescence due to alternate conformations of bound dye.^9−14^ Using a combination of two LCOs, qFTAA and hFTAA,^15^ we have previously discovered that different polymorphs appear to exist in plaque cores and periphery within the same plaque^9^ in transgenic APPPS1 mice rich in Aβ42.^16^ This difference was even more pronounced in APP23 mice, predominantly producing Aβ40.^17^ For both mouse models we observed a change in plaque morphology and staining pattern as the mice aged, which was referred to as plaque core maturation^9^ allegedly representing fibril polymorph rearrangement. Aβ-plaque polymorphism was also prolific when analyzing amyloid plaques in post-mortem AD patient samples from familial (fAD) as well as sporadic AD using the LCO technology.^18^ This study strongly suggested that a cloud-like diversity of Aβ conformations appears within each patient. In addition, using the same approach, we recently demonstrated that there was a difference in plaque structure between rapid progressing and slow progressing sporadic AD.^19^ Hence targeting specific polymorphs of Aβ aggregates is an attractive strategy for diagnostics and disease-modifying therapies for ADs. Considering recently approved monoclonal antibody drugs targeting Aβ-amyloids (Aducanumab and Lecanemab) and patient-specific response, it is important to understand Aβ turnover^20^ and its plausible dependency on fibril polymorphism.

The first transgenic AD mouse model was introduced in the mid-1990s^21^ based on the current understanding of the biochemical processing of the Amyloid-β precursor protein (AβPP) and how it is processed to form amyloid plaques. Since then, 197 mouse models of AD have been reported where of 77 are transgenic or knock-in for the AβPP gene and hence can be predicted to display Aβ plaque pathology.^22,23^ Mouse models of AD will also in the future be crucial in the further search for disease-relevant Aβ amyloid polymorphs.

The plaque-forming Aβ peptide exists in several different isoforms, predominantly ending at amino acids 38–43. The peptides are formed by the cleavage of AβPP by several endogenous proteases according to the amyloidogenic cleavage pathway.^3^ Most mouse models of AD pathology are based on a humanized AβPP gene flanked by familial mutations that promote the amyloidogenic processing pathway. The Swedish AβPP mutation KM670/671NL^24^ rendering overproduction of Aβ, is commonly used. Many mouse models are also combined with presenilin 1 (PS1) mutations to further exacerbate the Aβ42 production.

**Figure 1:**
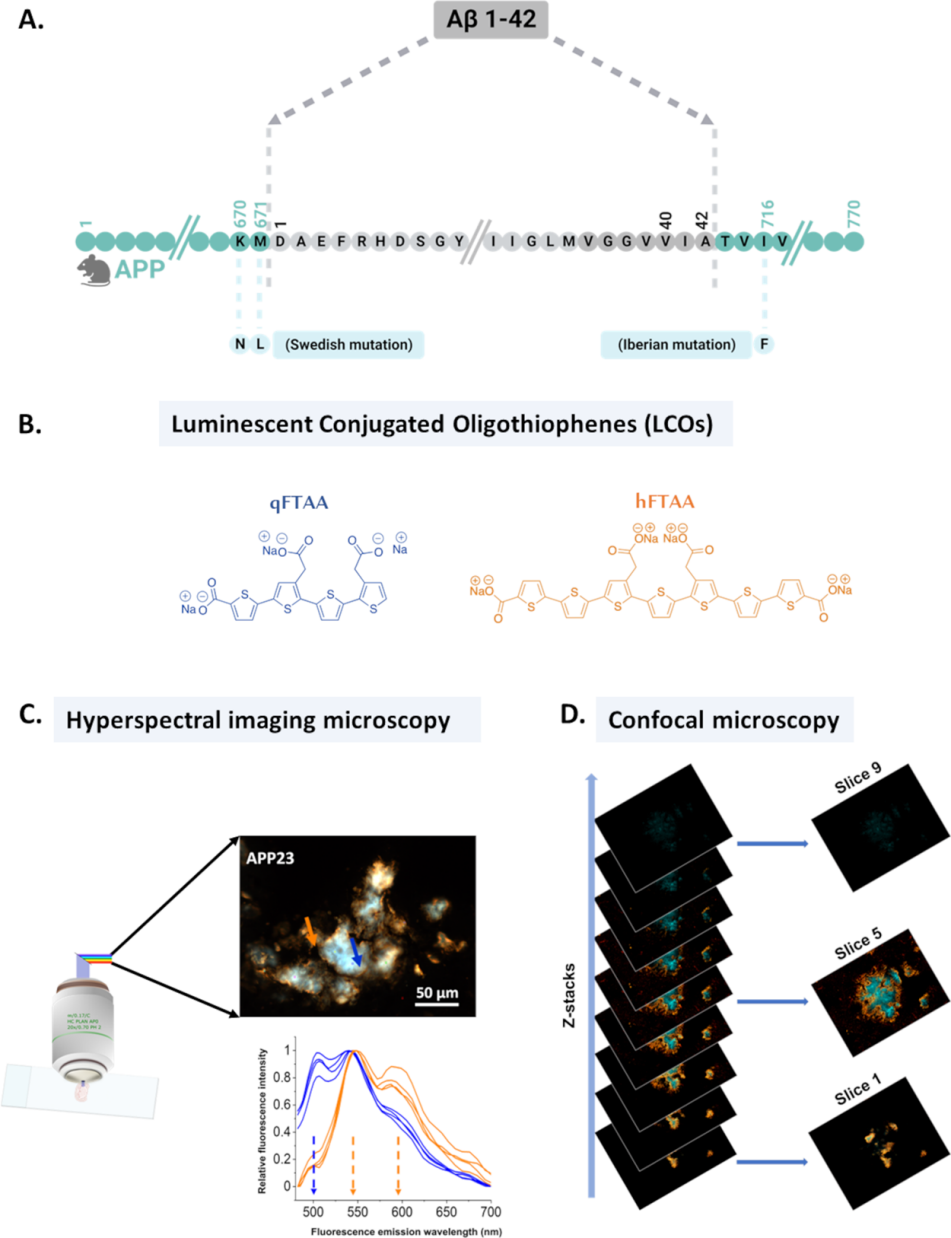
(A) Human AβPP protein highlighting the Swedish and Iberian disease mutants utilized to generate APP23, APPPS1, and *App^NL-F^* mouse models. (B) Thiophene-based conformation sensitive dyes: luminescent conjugated oligothiophenes (LCOs). (C) Hyperspectral fluorescence imaging of mouse brain section stained with LCOs: qFTAA and hFTAA, excited at 436 nm, and imaged using a long-pass emission filter. qFTAA and hFTAA give different emission spectra upon binding to amyloid fibril structures: qFTAA emits with a peak at 500 nm (blue arrow), while hFTAA emits with double peaks at 540 and 588 nm (orange arrows). (D) Z-stack confocal imaging of the mouse brain section that provides information from different sections of the plaque.

In previous studies, we found a relatively low abundance of Aβ-amyloids displaying qFTAA fluorescence in APPPS1 mice compared to APP23.^9^ This is likely a reflection of the lower qFTAA fluorescence we observed from in vitro formed recAβ1–42 fibrils compared to fibrils formed under the same conditions but from recAβ1–40.^9^ We, therefore, in this study compared three widely used mouse models with Aβ pathology expressing varying amounts of Aβ and Aβ42/Aβ40 isoform ratios (Figure 1A, Table S1). The transgenic APP23^17^ has a seven-fold overexpression of human AβPP with the Swedish mutation (KM670/671NL) and produces more Aβ40 than Aβ42. The transgene is expressed under the Thy1 promoter element, resulting in the production of AβPP mainly in neurons.^25,26^ APPPS1 is a transgenic mouse model with a 3-fold overexpression of human AβPP with the Swedish mutation. In addition, it expresses a PS1 variant (L166P) that elevates the Aβ42/Aβ40 ratio. Also in this mouse, the transgene is expressed under the Thy1 promoter element.^16^ In the *App*^*NL-F*^ knock-in model mouse, AβPP is expressed under the endogenous promoter, ensuring physiological levels of AβPP at cell type and temporally relevant locations. The Aβ sequence was humanized and the insertion of the Swedish and the Iberian mutations (I716F) led to a specific increase in Aβ42 production.

## Results and Discussion

### Analyzing Aβ Fibril Plaque Polymorphism by LCO Hyperspectral Microscopy

We have for several years analyzed amyloid fibril deposits of different proteins and used thiophene-based ligands,^27^ and other molecular scaffolds such as *trans*-stilbenes^28,29^ for optical assignment of distinct protein aggregates, i.e., amyloid fibril polymorphism on the folding and filament assembly levels.^7^ The notion behind this strategy is that different fibril polymorphs have different molecular structures of their ligand binding sites^7^ and should thereby be distinguishable by fluorescence properties of different dyes binding differently to these structures. Oligo-thiophenes with anionic side chains, LCOs, have been shown to be well-suited for amyloid detection due to high affinity and selectivity.^27^ The flexible structure of the LCOs allows these molecules to adapt their conformation to the shape of the binding site of the amyloid fibril. This property will alter the conformation and hence conjugation length of the bound LCO and afford different optical outputs depending on the fibril structure from one dye.

It is well established that prions manifest as different strains depending on the structure, i.e., the fibril polymorph, of the PrP amyloids and thereby display different incubation time, tissue tropism, and disease phenotype.^30^ The optical property of conformational sensitive LCOs was shown to enable the separation of different prion strains in studies of prion-infected transgenic tga20 mice. In other words, the infected tga20 mice displayed different LCO fluorescence from PrP amyloid deposits as a function of inoculated prion strain.^13^

We have in this study revisited our protocol of combining two LCOs to increase the contrast of LCO fluorescence spectra as a marker for Aβ-fibril polymorphism. The two LCO dyes, qFTAA (quadro-formylthiophene acetic acid) and hFTAA (hepta-formylthiophene acetic acid) (Figure 1B), show distinct spectral properties upon binding to different amyloid fibril structures.^15^ The dye qFTAA fluoresces with an emission spectrum peaking at around 500 nm upon binding to tightly packed bundled Aβ-fibrils.^31^ The dye hFTAA binds to both single filamentous and bundled Aβ-fibrils, with red-shifted emission spectra with peaks at 540 and 588 nm.^31^ We, therefore, employed co-staining with qFTAA and hFTAA as surrogate markers for amyloid polymorphism within Aβ amyloid plaque. It is known that Aβ amyloid plaque deposits have different microscopic morphologies when stained by immunohistochemistry and amyloid dyes. Aβ1–40 and Aβ1–42 amyloid fibril structural polymorphism is well documented by high-resolution structural techniques of fibrils formed in vitro,^32−36^ in purified human^8,37^ and mouse brain^38,39^ derived amyloid fibrils, and in seeding experiments using brain-derived fibrils as seeds for recombinant Aβ.^40,41^ The overall architecture is common among the fibril types. In-register parallel β-strands arranged in β-arches comprise the fibril filament cross-β-sheet structures. However, the fold, sequence arrangement of intermolecular interactions, protofilament packing, and fibril assembly appear dramatically different in the Aβ fibril polymorphs. If and how the Aβ fibril polymorphs are associated with AD onset and progression are currently not established.

It has been discussed that conformational variations, as reported by LCO staining, do not fully agree with the high-resolution cryo-EM structures of Aβ fibrils,^8^ in that LCO staining shows a wide variation of conformations in sAD^18^ while cryo-EM structures find one predominant (type I) filament structure in sAD.^8^ Furthermore, fAD also had one predominant polymorph (type II) according to,^8^ where LCOs showed a separation, while still highly variable, depending on the type of fAD.^18^ Interestingly *App*^*NL-F*^ and APP23 Aβ-amyloid filaments isolated and imaged by the same Cryo-EM procedure were reported to have the same main structure (type II).^8,39^ We therefore herein compared side by side Aβ-amyloid plaque conformational typing by LCO staining of three mouse models (Table S1, Figure 1A).

Co-staining with both qFTAA and hFTAA of plaques from aged (18 Mo) APP23 mice revealed two different fibrillar structural arrangements. Selected regions of interest (ROIs) from the core of the plaque were primarily occupied by qFTAA (blue-shifted spectrum with a peak at 500 nm) indicating tightly packed fibrils (Figure 1C, blue arrow). The core was surrounded by hFTAA-stained ROIs (red-shifted spectrum with peaks at 540 and 588 nm), proposing different polymorphs of fibrils in the periphery or corona of the plaque (Figure 1C, orange arrow). We supplemented the hyperspectral microscopy with confocal microscopy, allowing the use of multiple channels to utilize both the antibody and LCO staining at the same time, bringing out more detailed information about how the fibrils are organized in situ in different parts of an individual plaque (Figure 1D).

**Figure 2:**
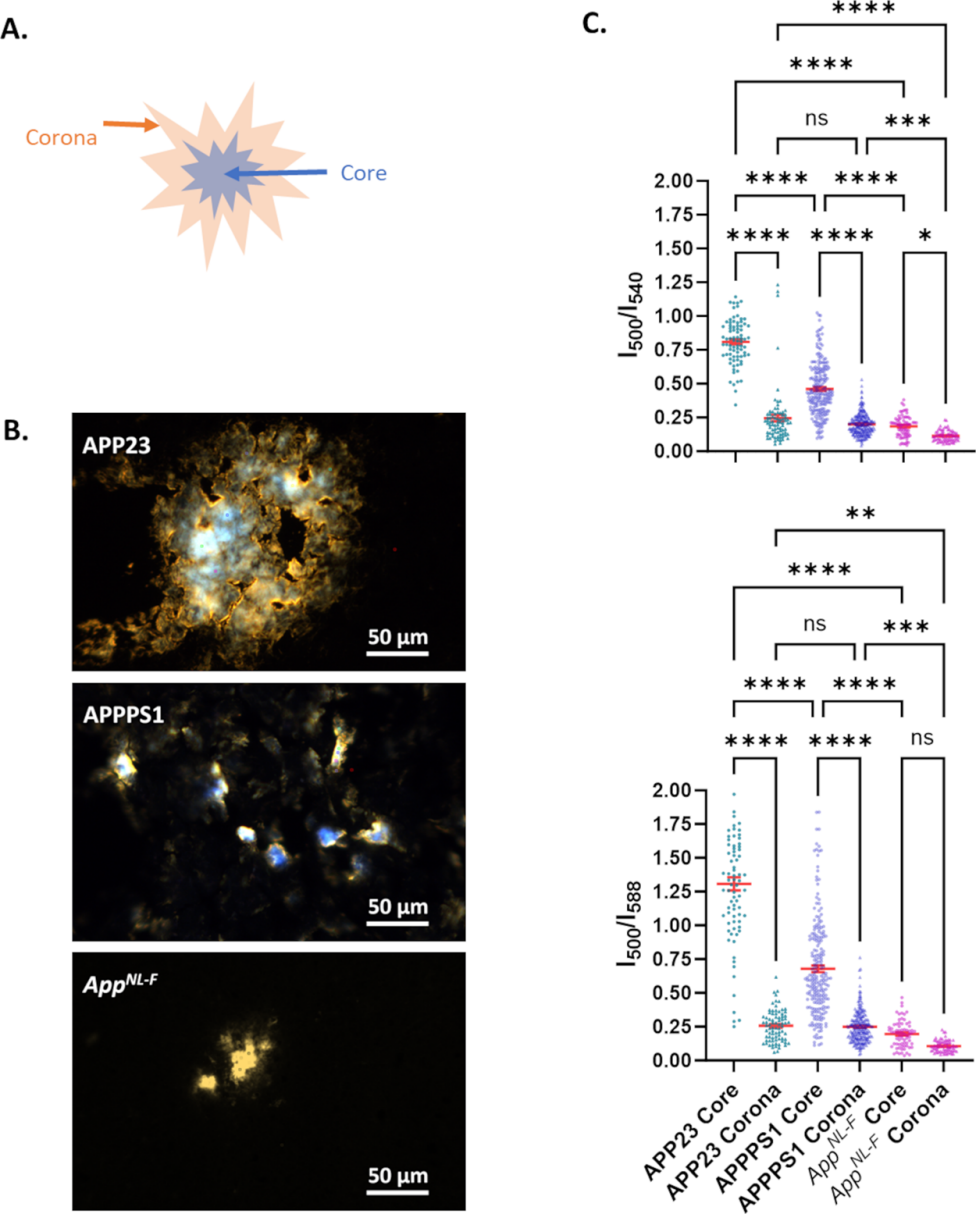
(A) Schematic representation of two distinct fibril polymorphic regions in plaques observed by double staining of qFTAA and hFTAA. The blue region represents the mature/bundled fibril-enriched plaque area termed the plaque core, dominated by qFTAA staining. The surrounding orange area of the plaque termed as corona is enriched with hFTAA-stained diffusely packed fibrils. (B) Hyperspectral image overview of plaques stained with qFTAA and hFTAA from APP23, APPPS1, and *App^NL-F^* mouse. The scale bars are 50 μm. (C) Fluorescence intensity ratiometric plot from the region of interest (ROI) from plaque cores and corona from APP23, APPPS1, and *App^NL-F^* mouse (aged 18−19 months). The error bars represent SEM. The upper panel shows the ratio of intensities at 500 and 540 nm (*I*_500_/*I*_540_), where 500 nm represents qFTAA emission and 540 nm represents hFTAA emission, respectively. In the lower panel, the intensity of 540 nm is replaced by that of 588 nm (*I*_500_/*I*_588_), which represents another hFTAA emission peak. An ordinary one-way ANOVA test was performed in GraphPad Prism for statistical analysis, where *p < 0.05; **p < 0.01; ***p < 0.001; and ****p < 0.0001 and ns = nonsignificant.

We then aimed for pairwise comparisons of Aβ-polymorphic differences between these commonly used mouse models expressing human AβPP. The analysis affords resolution of the organization of structures allowed by optical microscopy (∼1 μm) but with the advantages of selective molecular probing with LCOs and observing intact amyloid structures in their near-native environment using cryosections of the flash-frozen brain (Figure 2). We compared two AβPP-overexpressing transgenic mouse models (APP23 and APPPS1) but with different Aβ42/Aβ40 ratios (Table S1). We also compared AβPP knock-in model *App*^*NL-F*^ exhibiting endogenous AβPP-expression with a humanized AβPP transgene sequence with the overexpressor APP23. Using our established protocol^9,18,19^ for the LCO discrimination of Aβ polymorphism as well as antibodies against different epitopes of the Aβ peptide to discriminate the two isoforms we deduced structural differences and how they corresponded to expression and dominating Aβ species.

First, age-matched APP23 and APPPS1 mouse brain sections (18 and 19 months, respectively) were stained with a combination of qFTAA and hFTAA and full fluorescence spectra were collected using hyperspectral epifluorescence microscopy.^42^ Four regions of interest (ROIs), each comprising 5 × 5 pixels (corresponding to ∼1 × 1 μm), from the core (Figure 2A,B) and 4 ROIs from the corona (Figure 2A,B) of the plaques were analyzed from each plaque. In total, 15 images comprised 20 plaques for the APP23 mouse, and 18 images contained 54 plaques for APPPS1 were analyzed. Fluorescence intensity ratiometric analyses were performed by division of the fluorescence intensity at 500 nm (qFTAA) with the fluorescence intensity at 540 and 588 nm (hFTAA) for each ROI (I_500_/I_540_ and I_500_/I_588_). We first compared the APP23 and APPPS1 mice. The analysis revealed a higher abundance of qFTAA fluorescence in the plaque cores of the APP23 model compared to that of APPPS1. In both mouse models, the qFTAA fluorescence was higher in the core compared to the corona (Figure 2C). This demonstrated that different transgenic genotypes have different fibril structures in the plaques depending on transgenic genotype and that the morphology differs between different parts (core and corona) of the same plaque (Figure 2C).

The results were coherent with our previous data.^9^ Both these models overexpress Aβ but the Aβ42/Aβ40 ratio is different in that APP23 largely generates Aβ40 while APPPS1 has up to 4.3-fold excess of Aβ42^43^ (Table S1). To delineate if total Aβ load or Aβ variant is the dominating denominator of polymorphic structure, we compared the APP23 and APPPS1 mice with *App*^*NL-F*^ mice, again analyzing 19 plaques from 15 images of 18-month-old mice. *App*^*NL-F*^ mice are known to generate almost exclusively Aβ42, whereas the AβPP expression is at endogenous levels (Table S1).^44^ The *App*^*NL-F*^ mice exhibited lower qFTAA fluorescence in both core and corona than the other two mouse models with the most striking difference between the three genotypes being observed in the plaque cores (Figure 2C).

### Aβ Fibril Plaque Polymorph Development during Aging

**Figure 3:**
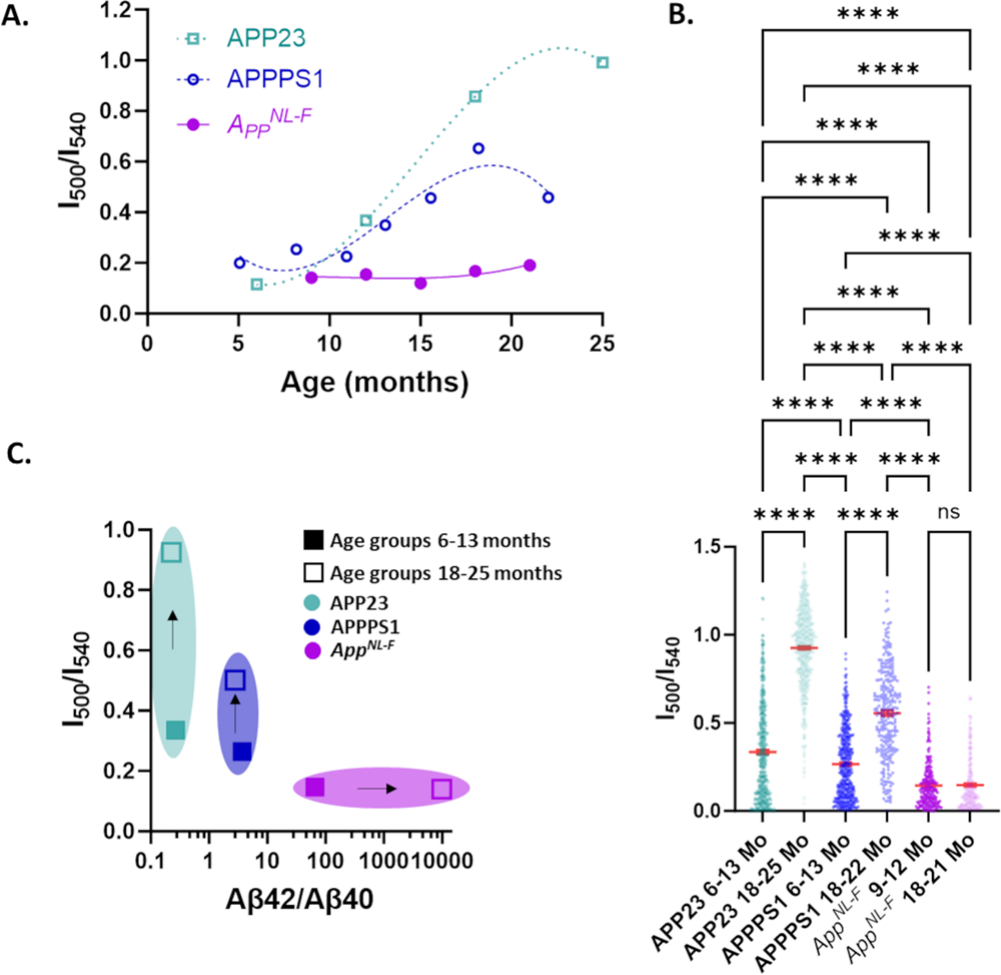
(A) Comparison of the ratio of the fluorescence intensity of the emitted light at 500 and 540 nm, *I*_500_/*I*_540_, of plaque cores versus mouse age of the *App^NL-F^* mouse groups (filled circle, solid line) with APP23 (open squares, dotted line) and APPPS1 (open circle, dashed line published in Nyström et al. 2013.^9^ The lines are a third-order polynomial fitting of mean values of the intensity ratio versus age to show the trend. For APP23 raw data from 5 mice at 6 months, 7 mice at 12 months, 5 mice at 18 months, and 5 mice at 25 months of age were analyzed. APPPS1 raw data are compiled from a total of 19 mice.^9^*App^NL-F^* raw data comprises 3 mice at 9 months, 1 mouse at 12 months, 1 mouse at 15 months, 1 mouse at 18 months, and 3 mice at 21 months. (B) Statistical analysis of the mice groups as a comparison of young (<13 months) and old (>18 months) age. The error bars represent SEM. An ordinary one-way ANOVA test was performed in GraphPad Prism for statistical analysis, where ****p < 0.0001 and ns = nonsignificant. Note that the imaged Aβ-aggregates in 6-month-old APP23 mice appeared to be intracellular inclusions and not bona fide Aβ-amyloid plaque cores. (C) Diagram showing the comparison of the ratio of intensity *I*_500_/*I*_540_ of the plaque core of young and old mice versus Aβ amyloid content in those mice as Aβ42/Aβ40 ratio (Aβ concentrations from refs (43−45)) which indicates that an elevated Aβ42/Aβ40 ratio corresponds to the high abundance of hFTAA fluorescence. Arrows are directed from young to old mice.

*App*^*NL-F*^ mouse brain had a very low abundance of qFTAA-positive plaques at 18 months. Differential qFTAA/hFTAA staining of plaque core versus corona developed during aging in both APPPS1 and APP23 mice, where increased qFTAA fluorescence of the plaque core as a function of mouse age with a transition at 12 months was reported by us as plaque core maturation.^9^ At very old age (>18 months) we previously observed a drop in plaque core qFTAA positivity due to increased hFTAA staining in APPPS1 mice.^9^ We therefore moved on to analyze the qFTAA/hFTAA ratio over several *App*^*NL-F*^ mice ages, 9–21 months (Figure S1A). We did not observe a noticeable trend of altered qFTAA fluorescence with *App*^*NL-F*^ mouse age (Figure 3A). The *I*_500_/*I*_540_ ratio for the *App*^*NL-F*^ mouse was around 3-fold lower compared to that of the previously published aged matched APPPS1 mouse at 21 months (Figure 3A). Our previously published data on APPPS1 mice using the same method on the contrary showed a clear transition toward a more densely packed amyloid in the core of the plaques at ∼12 months, reflected by the increase in qFTAA fluorescence^9^ (Figure 3A) and subsequent decrease >18 months,^9^ as discussed above. We therefore here performed a more complete analysis also of APP23 plaque cores as a function of mouse age between 6 and 25 months to include in the comparison with *App*^*NL-F*^ (Figure S1B). APP23 plaque cores showed a very distinct transition above 12 Mo (Figure 3A). APP23 plaque cores showed elevated *I*_500_/*I*_540_ ratios being even higher after 18 months than APPPS1 (Figure 3A) making the discrepancy between *App*^*NL-F*^ mice and APP23 even stronger than for APPPS1 (Figure 3A). Statistical analysis of the separate age groups showed significant differences between all genotypes, young <13 Mo as well as older mice >18 Mo (Figure 3B).

As eluted to above, when analyzing the distribution of the qFTAA/hFTAA fluorescence ratio of the plaque cores we observed a trend in fluorescence signal tilting toward low qFTAA and high hFTAA positivity that corresponded to increased Aβ42/Aβ40 ratio. To test this hypothesis, we plotted the Aβ42/Aβ40 ratio versus qFTAA/hFTAA fluorescence ratio for the mouse groups in the study at young (6–13 Mo) and old (>18 Mo) ages. While the qFTAA/hFTAA comparison was limited to plaque cores and the Aβ peptide amyloid content was from brain homogenate^43−45^ it appeared to correlate as hypothesized. The discrepancy, while significant in young mice, was augmented during aging of the three genotypes (Figure 3C).

### Unbiased Image Analysis of Hyperspectral Images

**Figure 4:**
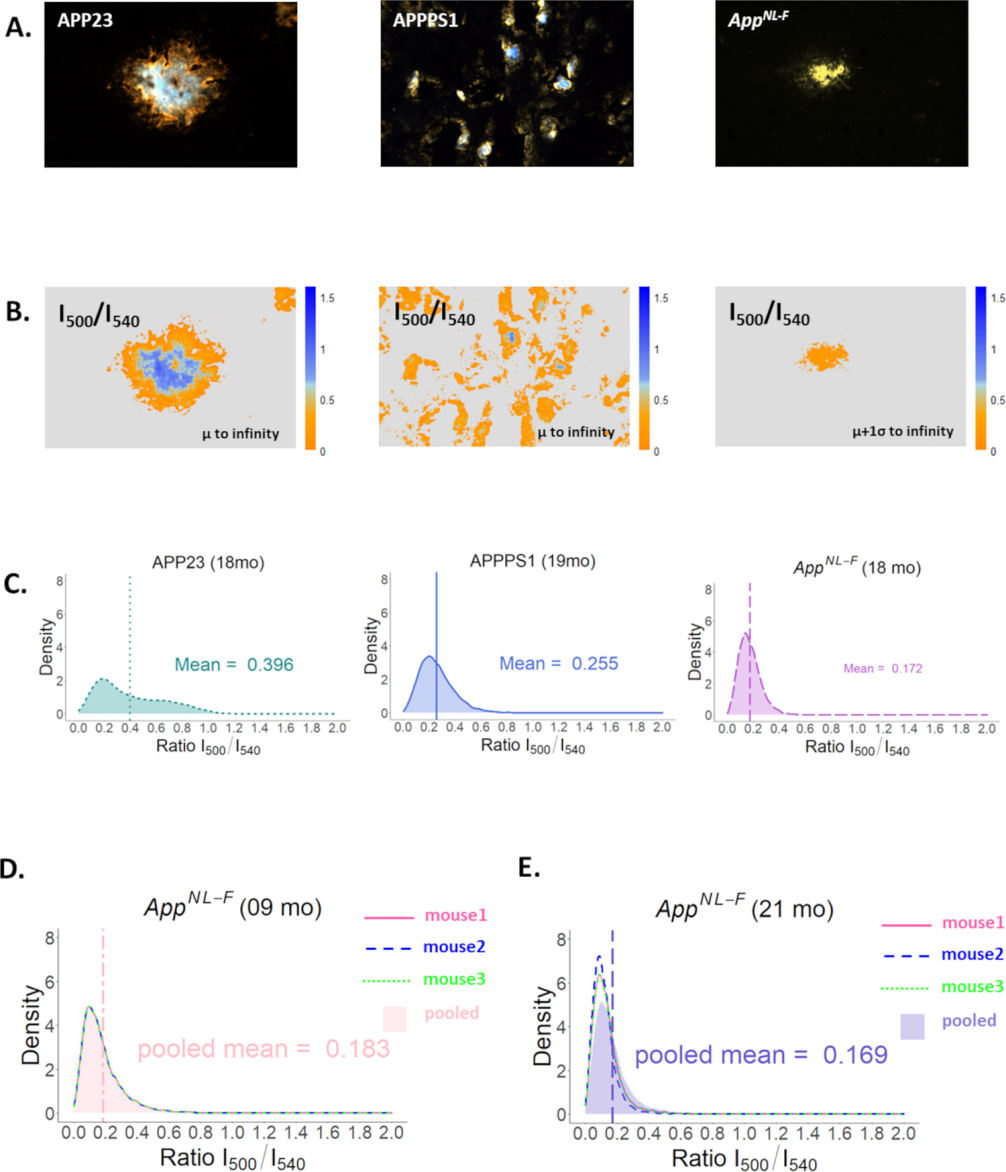
(A) Representative hyperspectral images of plaques from APP23, APPPS1, and *App^NL-F^* mice stained with qFTAA and hFTAA. (B) Representation of the same plaques as heat maps after unbiased whole image analysis applying the optimal filter setting for these three genotypes to remove the unwanted signals or intensity at an intensity ratio of *I*_500_/*I*_540_. (C) Pixel density distribution plots for the corresponding genotypes, calculated from the intensity ratio matrix at *I*_500_/*I*_540_. For each density plot, a total of 10 images from one mouse from each genotype (aged 18–19 months) were analyzed. (D) Pixel density distribution plots for *App^NL-F^* mouse calculated at intensity ratio *I*_500_/*I*_540_ at 9 months of age from 3 individual mice (represented by lines) and their pooled intensity ratio at *I*_500_/*I*_540_ (represented by the pink shade). (E) Pixel density distribution plots for *App^NL-F^* mouse calculated at intensity ratio *I*_500_/*I*_540_ at 21 months of age from 3 individual mice (represented by lines) and their pooled intensity ratio at *I*_500_/*I*_540_ nm (represented by the violet shade).

The image analysis above was based on ocular determination of the core and corona. Similar numbers of 5 × 5 pixel ROIs (∼1 × 1 μm) were collected from both core and corona regardless of size or number of plaques in each specific genotype. However, the size and number of plaques differ largely between the mouse models. APP23 mice exhibit large plaques as well as CAA (not analyzed here), while the plaques in APPPS1 mice are smaller and more abundant. The knock-in *App*^*NL-F*^ mice, expressing endogenous amounts of AβPP present both fewer and smaller plaques than the overexpressing models, as can be expected. To get an overall score of qFTAA versus hFTAA positivity of the Aβ amyloid, we performed an unbiased whole image analysis that considers each pixel of the hyperspectral images collected. This analysis method will not report on the region-specific differences of plaque morphology (core and corona) but rather on the overall proportion and variability of amyloid staining within each image. This image analysis (described in Supporting Methods and Figures S2 and S3A–D) can be performed at any wavelength ratio and here we used *I*_500_/*I*_540_ and *I*_500_/*I*_588_ as in the ROI analysis. Our ROI-based results showing that APP23 demonstrated the highest and *App*^*NL-F*^ mice the lowest abundance of qFTAA positivity (Figure 2C) was confirmed in this unbiased image analysis (Figure 4A–C). APP23 showed considerably higher *I*_500_/*I*_540_ and *I*_500_/*I*_588_ ratios and a wide distribution compared to APPPS1 (Figure S3E). *App*^*NL-F*^ mice showed the lowest ratios and the narrowest distribution (Figure S3E). Density plots comprising the density of hyperspectral image pixels of qFTAA/hFTAA *I*_500_/*I*_540_ ratios were generated to visualize and compare genotypes, individuals, and within age groups (Figure 4A–E). This procedure was performed for all three models at 18–19 months. The mean ratio for aged mice was 0.396 for APP23, 0.255 for APPPS1, and 0.172 for *App*^*NL-F*^ (Figures 4C and S4B). The density plot of different age groups of *App*^*NL-F*^ mice at the intensity ratio matrix at *I*_500_/*I*_540_ also did not show any significant individual differences (Figure 4D,E) or differences at different ages (cf. Figure 4D,E), all the age groups are essentially overlapping (Figure S4A). These results implied that there were no significant changes in the plaque structure over time.

### Polymorphic Properties of Pure Fibrils of Aβ1–40 versus Aβ1–42

**Figure 5:**
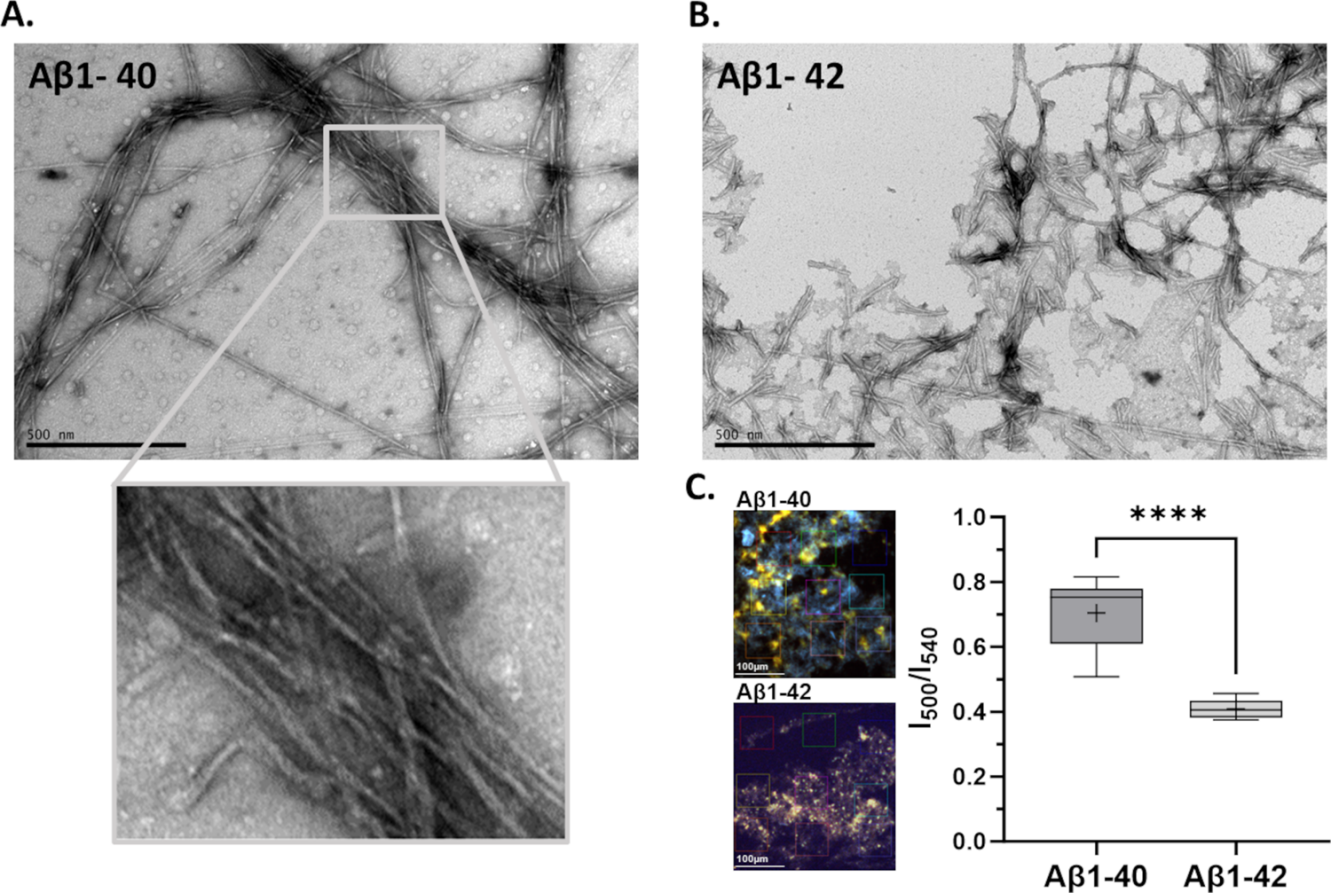
Negative stain TEM images and hyperspectral fluorescence analysis of recombinant Aβ1–40 and Aβ1–42 fibrils fibrillated at 10 μM in PBS buffer pH 7.4 at 37 °C without shaking. (A) TEM image of Aβ1–40 fibrils at the end of the fibril growth phase (10 h). The fibrils are very long and form laterally associated bundles of intertwined fibrils (zoom-in box). (B) TEM images of Aβ1–42 fibrils at the end of the fibril growth phase (3 h) are shorter and predominantly solitary. Scale bars 500 nm. (C) Recombinant fibrils at the end stage (25 h) stained simultaneously with qFTAA and hFTAA, deposited on microscope slides, and analyzed by hyperspectral microscope in analogy with analysis of mouse tissue samples. Scale bar 100 μm. Higher *I*_500_/*I*_540_ ratios are observed for Aβ1–40 compared to Aβ1–42 fibrils. Unpaired *t* test was performed for statistical analysis, where ****p < 0.0001.

Aβ fibrils generated in vitro render different packing architectures. Aβ1–42 fibrils are predominantly solitary, albeit clustered, while Aβ1–40 tends to form thick laterally assembled bundles comprising several fibril filaments (Figure 5A,B). Tightly packed Aβ-fibrils will render a higher qFTAA signal in in vitro experiments^9,31^ and Aβ1–42 fibrils are to a lower degree than Aβ1–40 fibrils associated with high qFTAA fluorescence (Figure 5C). Hence, these in vitro results indicate that the fibrils are arranged differently in vivo in the plaque core region compared to the corona. It also indicates that the Aβ variant composition (Aβ40 vs Aβ42) can influence the plaque structure of the different mouse models. The data support that APP23 plaque cores have more tightly packed fibrils compared to APPPS1 and *App*^*NL-F*^ plaque cores.

### Immunofluorescence of Aβ Plaque

**Figure 6:**
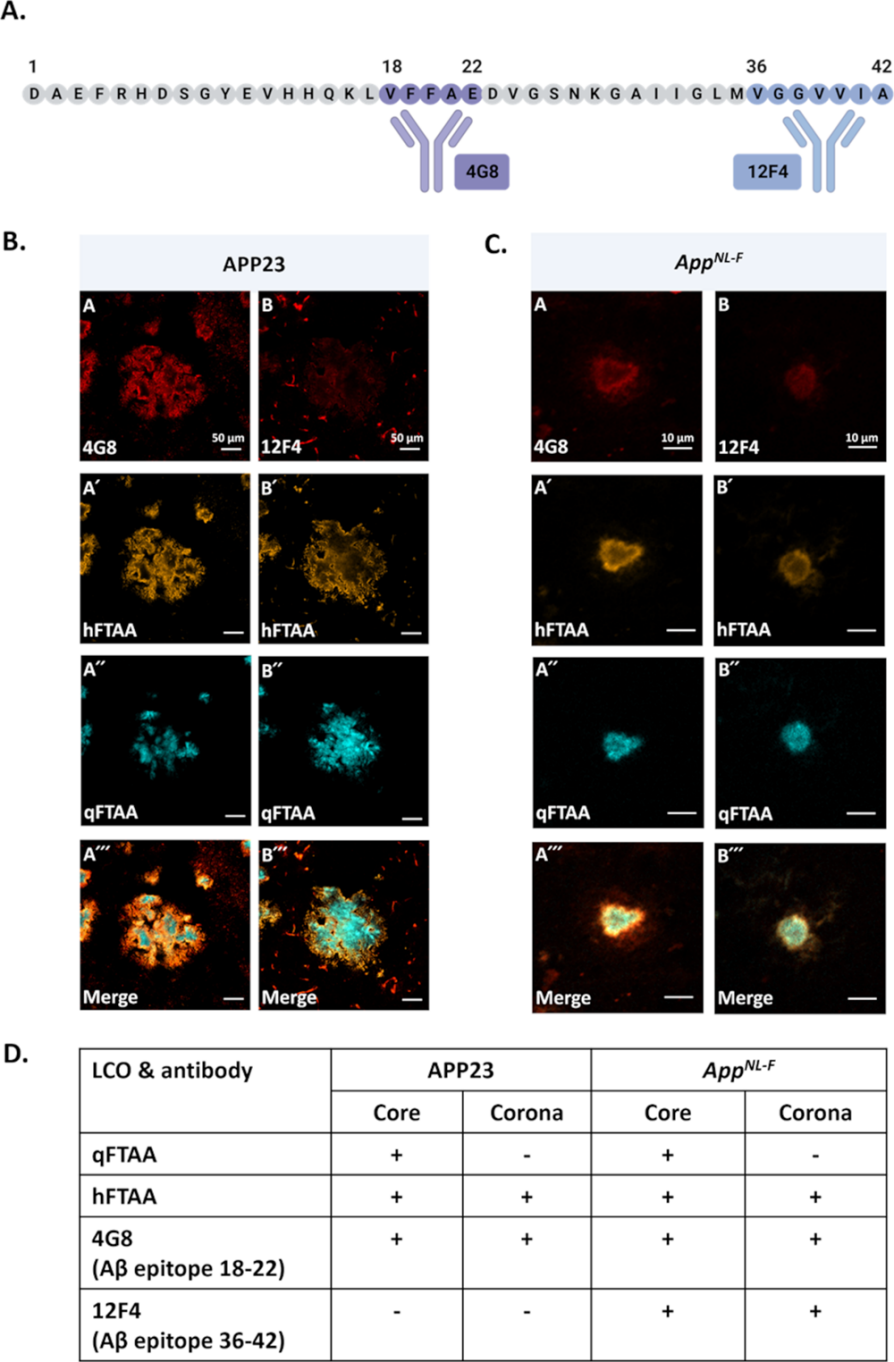
(A) Schematic representation of the 4G8 and 12F4 antibodies. The 4G8 antibody recognizes the Aβ epitope sequence 18–22; meanwhile, the 12F4 antibody recognizes the Aβ epitope sequence 36–42. (B) Antibody and LCO co-staining of plaque from APP23 mouse. Panel A shows 4G8 antibody staining, panel A′ shows hFTAA staining, panel A″ shows qFTAA staining and panel A‴ shows the merged view of all three stains. Panel B shows 12F4 antibody staining, panel B′ shows hFTAA staining, panel B′′ shows qFTAA staining and panel B′′′ shows the merged view of all these three staining. The scale bars are 50 μm. (C) Antibody and LCO co-staining of plaque from *App^NL-F^* mouse. Panel A–A‴ and B–B″′ show similar antibody and LCO co-staining as shown in (B). Note that the scale bars in (C) are 10 μm. Images in (B) and (C) are the single focal planes from the *z*-stack images in the confocal microscope where all the channels have a maximum signal. (D) summary of LCO and antibody staining in different plaque regions in APP23 and *App^NL-F^*, respectively.

To further verify the molecular basis for the Aβ-fibril polymorphism in vivo, co-staining with antibody and LCO was performed, and imaged by confocal microscopy. We here focused on the two mouse model extremes in the qFTAA/hFTAA ratio and Aβ42/Aβ40 ratio, APP23 and *App*^*NL-F*^. The monoclonal antibody 4G8 (Aβ epitope 18–22) was used as a pan-Aβ detector, while the antibody 12F4 (Aβ epitope 36–42) was used to selectively stain Aβ42 (Figure 6A). The antibody-LCO co-staining revealed that APP23 plaque cores have qFTAA binding along with hFTAA and 4G8 antibodies (Figure 6B). The periphery or corona showed only hFTAA and 4G8 binding (Figure 6B). 12F4 is poorly bound to the outermost part of the corona (Figure 6B). This co-staining showed that individual APP23 plaque comprised two distinct fibril polymorph regions of core and corona. The plaque core consists predominantly of compact Aβ40 fibrils; meanwhile, the corona consists mainly of diffusely packed Aβ40 fibrils. Very little Aβ42 appeared to be present in the APP23 Aβ-plaque as deduced from immunofluorescence. This could be the result of hidden 12F4 epitopes of Aβ42. Nonetheless, the low amount of Aβ42 within plaque is coherent with previous mass spectrometry data for cored plaques in the similar mouse model APP_swe_,^46^ showing exceptionally low Aβ42/Aβ40 ratios, likely undetectable by immunofluorescence herein. Interestingly, the low abundance of Aβ42 compared to Aβ40, positively correlated with higher qFTAA/hFTAA ratios within the core plaque of APP_swe_ mice^46^ supporting the correlation we have eluted to in this discussion.

For *App*^*NL-F*^ mice, qFTAA positivity was essentially not observable using epifluorescence hyperspectral microscopy (Figures 2 and 3). However, with confocal microscopy and optical sectioning, we detected qFTAA positivity in tiny cores (Figure 6C) of approximately 80% of the plaques. The small size of the cores is likely the reason for a few areas with elevated *I*_500_/*I*_540_ ratios, rendering an overall elevation of the ratio of core versus corona also for *App*^*NL-F*^ mice (Figure 2C). Notably, the cores do not significantly change with *App*^*NL-F*^ mouse age (Figures 3A and 4D,E). This demonstrates that the qFTAA positive part of each plaque is minute in comparison to >18 Mo APP23 where it is a dominating species. Co-staining with antibodies revealed that the plaque corona displayed hFTAA, 4G8, and 12F4 binding but no qFTAA fluorescence (Figure 6C) and the core was positive for hFTAA, qFTAA, 4G8, and 12F4. Thus, we conclude that the qFTAA-positive tiny plaque cores in *App*^*NL-F*^ are composed of compact Aβ42 fibrils, whereas most Aβ-plaques and the corona consist of diffusely packed Aβ42 fibrils. Hence, since qFTAA can stain *App*^*NL-F*^ cores, essentially devoid of Aβ40, it appears that qFTAA can also bind to subtypes of tightly packed Aβ42 fibril polymorphs. We have previously observed elevated qFTAA fluorescence in transgenic *Drosophila* with tightly packed Aβ1–42 fibrils expressed in glial cells compared to intraneuronal ring-tangle-like aggregates expressed in neurons.^47^ The staining profiles of aged APP23 and *App*^*NL-F*^ plaque are summarized in Figure 6D.

### How Do Aβ Plaque Structures in Mouse Models Relate to Human Alzheimer’s Disease?

The amyloid strain phenomenon and its dependence on fibril conformation have been extensively explored in the context of the prion protein and prion disease, where it is established that the prion structure correlates with disease phenotype.^30^ Amyloid fibril polymorphism coherent with what is known for prion strains appears evident for Aβ and AD.^48,49^ The complexity of Aβ aggregation and the consequent heterogeneity of Aβ amyloid fibrillar structures was recently reviewed.^50^ Aβ-amyloid deposits as both plaques^5^ and cerebral amyloid angiopathy (CAA)^51^ and can be found in asymptomatic older individuals. Many individuals never develop AD or other dementias, although they reach old age. It is hence important to delineate the molecular details of the Aβ amyloid fibril polymorphs to better address the progression of Aβ amyloidosis and the distribution of benign and disease-relevant fibril types with molecular diagnostic and therapeutic strategies. The molecular tracers used in vivo in clinical practice today do not readily distinguish between disease-related and nondisease-related amyloid deposition.^52^ Furthermore, not all disease-associated Aβ amyloids are detected by amyloid PET tracers. For example, carriers of the Arctic mutation (E22G), despite extensive Aβ fibril load, do not retain PiB in PET imaging.^53^ A wide distribution of LCO staining patterns in different patients suggests that the polymorphic patterns of different plaques are very hard to predict.^18^ How Aβ amyloids form and evolve over time is important to understand disease progression, diagnostics, and treatment.

In this work, we aimed to further understand the influence of isoform and expression level of the Aβ peptides on amyloid fibril polymorphism in different AβPP mouse models by direct staining and imaging of Aβ plaque in situ. We found that Aβ plaques in the three mouse models included in this study exhibit several different morphologies. The Aβ plaque morphology changes over time in APP23 and APPPS1 mice^9^ but not in *App*^*NL-F*^ mice. *App*^*NL-F*^ mice contain small plaques dominated by hFTAA fluorescence. Plaque formation onset has been reported at 6 weeks of age in APPPS1 mice^17^ and at 6 months of age in both APP23^17^ and *App*^*NL-F*^(44) mice. This indicates that the age of the mouse or the age of the plaque cannot exclusively explain the difference in plaque morphology and fibril structure between APP23 and *App*^*NL-F*^ mice. Notably, *App*^*NL-F*^ and APP23 Aβ-amyloid filaments isolated and imaged by cryo-EM were reported to have the same main structure polymorph (type II).^8,31^ While it is possible that LCO fluorescence is influenced by factors such as other proteins/glycans/lipids bound to the amyloid fibrils, the pure in vitro data (Figure 5) strongly support that the LCO fluorescence mainly reports on fibril polymorphism. It can however not be ruled out that variable nonamyloid composition of the different plaque types influence morphology and LCO fluorescence of the proteins within the amyloid deposits. To understand the discrepancy with cryo-EM, it is conceivable that LCO staining reports on higher-order assemblies of filaments with the same filament fold, or that cryo-EM preparation, isolation, and selective particle imaging influence the cryo-EM results. The latter effect has recently been discussed in in situ cryo-EM tomography of *App*^*NL-G-F*^ mice compared with cryo-EM structure determination of isolated ex vivo fibril material.^54^ The structures of fibrils within densely packed plaque cores are likely not resolved in the reported single-particle cryo-EM studies. It is also noteworthy that the type II filament structure reported for 21-month-old APP23 mice was composed of Aβ42,^39^ suggesting that this structure corresponds to peripheral fibrils of the corona or smaller fibrils within the brain, not being intrinsic parts of amyloid plaque.

Several PS1 mutations found in human fAD act by increasing the release of Aβ peptides from AβPP. However, the Aβ isoform varies between mutations. PS1-A431E results in a high abundance of Aβ peptides but a low Aβ42/Aβ40 ratio (approximately 1/7th for the mutant carrier compared to the average for cases of sporadic AD in the same study).^55^ On the contrary carriers of PS1-E280A in a different study had an almost doubled Aβ42/Aβ40 ratio compared to sporadic AD cases^56^ (both studies used ELISA to deduce the Aβ42/Aβ40 ratio). In other words, carriers of PS1-A431E generate more Aβ40, and PS1-E280A carriers generate more Aβ42 than patients without the mutation. In this sense, APP23 mice are like PS1-A431E while *App*^*NL-F*^ resembles PS1-E280A in terms of dominating aggregated Aβ peptide isoform. In studies of human AD cases, it has previously been shown that Aβ plaques in PS1-E280A carriers display very low qFTAA fluorescence (low intensity at 500 nm) while PS1-A431E carriers generate plaques with high qFTAA signature.^18^ This is consistent with the LCO signatures of aged APP23 and *App*^*NL-F*^ mice described here. We hence conclude that time is an important factor for the generation of the tightly packed cored plaques seen in APP23 mice and that this cored structure is promoted by the presence of abundant Aβ40. The process of age-dependent plaque core rearrangement called plaque maturation of APPPS1 and APP23 mice is poorly understood but appears to entail tight lateral packing of multifilamentous fibrils. This process would be very interesting to study by high-resolution methods such as in situ cryo-EM tomography at different ages of mice in conjunction with LCO staining, as was performed in this study. Plaque core maturation towards high qFTAA fluorescence is a feature much less pronounced in *App*^*NL-F*^ mice forming almost exclusively Aβ42 plaque. Hypothetically if *App*^*NL-F*^ mice were to be aged for a very long time >30 Mo it is conceivable that the qFTAA signature would increase. Also, the expression levels generating more Aβ in APP23 and APPPS1 mice than in *App*^*NL-F*^ mice may matter. However, this is not the full explanation. A previous study treating APPPS1 mice with a BACE-1 inhibitor decreased total Aβ production and hence the number and size of Aβ-amyloid plaque in young mice, but the qFTAA/hFTAA ratio was altered by increasing in cortical regions and decreasing in the thalamus, hypothalamus, and hindbrain regions.^57^ No difference was seen in treated versus nontreated 14-month-old APPPS1 mice. These results indicate that the issue is more complex than merely Aβ-concentration.

## Concluding Remarks

The LCO-hyperspectral approach for amyloid imaging allows mapping of the spatial distribution and the substructural organization of nearly intact amyloid structures in situ in their near-native environment of formation. Our temporal studies of differential development of Aβ-amyloid polymorphs in various AβPP expressing mice, considering the variable human Aβ-pathology, strengthen the argument for translational work on using various mouse models as valuable prototypes for mapping Aβ-fibril polymorphism.

## Methods

### Animals

All animal experiments were conducted in agreement with protocols approved by the local Animal Care and Use Committees, respectively. Animal experiments at Linköping University were approved by the animal ethics committee (#10925-2020, #13028-2021). *App*^*NL-F*^ mice were reared by Takashi Saito and Takaomi Saido laboratories at the RIKEN Center for Brain Science, Tokyo, Japan. APP23 and APPPS1 mice were reared at Mathias Jucker lab at Hertie Institute for Clinical Brain Research, Tübingen, Germany. APP23 mouse tissues were handled as described previously.^43^ APPPS1 mouse tissues used here are described previously.^9^ This study hence allowed a direct comparison of *App*^*NL-F*^ and APP23 with previous data of APPPS1. Data from a total of 50 brains were included in the study: APPPS1 (*n* = 19), APP23 (*n* = 22), and *App*^*NL-F*^ (*n* = 9).

### Preparation of Tissue Sections for Fluorescence Microscopy

Flash-frozen mouse brains of transgenic APP23 and APPPS1 and knock-in *App*^*NL-F*^ were used for making brain cryosections of 10 μm. Cryosections were fixed in two consecutive ethanol concentrations of 96% (v/v) and 70% (v/v), 10 min for each concentration at room temperature. Tissue sections were further rehydrated with dH_2_O and PBS, pH 7.4, each step having 10 min of incubation time. Following the rehydration steps, tissue sections were incubated with LCOs (2:1 qFTAA and hFTAA, prepared as described below) for 30 min. After incubation with LCOs, tissue sections were washed with PBS 3 times and incubated in PBS for 5 min. Tissue sections were then dried in ambient air and mounted with DAKO fluorescence mounting medium and coverslip #1. The LCOs (qFTAA and hFTAA) were synthesized as described earlier.^15^

LCOs were dissolved in 2 mM NaOH in dH_2_O to have a stock solution of 1 mg/mL, which gives a qFTAA solution of 1.8 mM and an hFTAA solution of 1.1 mM. LCOs were stored at 4 °C until further use. For double staining of mouse brain sections with LCOs, qFTAA was diluted to 1:10000 and hFTAA 1:1392 and mixed in a ratio of 2:1 correspondingly. This gives a final staining solution of 120 nM qFTAA and 262 nM hFTAA.

For antibody staining, 4G8 (Aβ epitope 18–22) and 12F4 (Aβ epitope 36–42) antibodies were diluted to 1:300 to stain mouse brain sections, followed by Alexa Fluoro 594 diluted to 1:400. For co-staining of antibodies and LCOs, the flash-frozen tissue sections were fixed at 70% (v/v) ethanol at 4 °C for 3 min. Prior to 70% (v/V) ethanol incubation, tissue sections were kept at room temperature for 30 min. Following ethanol incubation, tissue sections were rehydrated in dH_2_O for 2 × 2 min and in PBS for 10 min. Later tissue sections were blocked with 5% goat serum in PBS-T (0.1% triton x-100) at room temperature for 1 h. Tissue sections were then incubated with 4G8 and 12F4 primary antibodies overnight at 4 °C. After primary antibody incubation, tissue sections were washed with PBS-T for 3 × 10 min. Following the washing steps, tissue sections were incubated with Alexa Fluoro 594 secondary antibody at room temperature for 1 h. Later tissue sections were washed with PBS for 3 × 10 min. Then tissue sections were subsequently incubated with LCOs and mounted as described above.

### Hyperspectral Fluorescence Microscopy

For hyperspectral imaging of mouse brain sections, a LEICA DM6000 B microscope was used and equipped with a spectral camera (Applied Spectral Imaging, Israel). A 436 nm long pass excitation filter (436/10 (LP475)) was used for image acquisition. Images were acquired with a 20× objective. Images were acquired with 20× objective except for the mouse groups of *App*^*NL-F*^ at different ages (9, 12, 15, 18, and 21 months). These images were captured with a 40x objective. The age series of *App*^*NL-F*^ mice comprised a total of 9 mice (*n* = 3 at 9 Mo, *n* = 1 at 12 Mo, *n* = 1 at 15 Mo, *n* = 1 at 18 Mo, and *n* = 3 at 21 Mo). For the age comparison of APP23, we analyzed a total of 22 mice (*n* = 5 at 6 Mo, *n* = 7 at 12 Mo, *n* = 5 at 18 Mo, and *n* = 5 at 25 Mo). At 6 Mo mostly intracellular inclusions were present in APP23. For age comparison of APPPS1 data reported by us previously were plotted from,^9^ comprising a total of 19 mice.

Representative images from each genotype were analyzed. From each image, four regions of interest (ROI) were selected from the core and 4 ROIs were selected from the corona of each plaque. Fluorescence intensity at 500 and 540 or 588 nm of the spectra from each ROI was used to generate the intensity ratiometric plots *I*_500_/*I*_540_ or *I*_500_/*I*_588_ respectively. Representative images from each genotype were used for side-by-side analysis and comparisons in Figures 2 and 4.

### Confocal Microscopy

Zeiss LSM780 confocal microscope was used to acquire z-stack images of LCO and antibody-co-stained tissue sections. Argon 458, 488, and 514 nm laser lines and DPSS 561-10 laser lines were used to excite the LCOs and Alexa Fluoro 594. Images were acquired with Plan-Apochromat 20*x*/0.8 M27 objective with a frame size of 1024 × 1024 pixels and scanning area at zoom 1.0.

### Unbiased Image Analysis in RStudio

Using an in-house generated program in RStudio, the ratio between intensity at 500 and 540 nm in each pixel was calculated. Pixels containing only background fluorescence were filtered out by the program (Figure S2A–D). Fluorescence emission intensity from hyperspectral imaging data for all pixels on an image at 500, 540, and 588 nm respectively were exported as text files using the spectra view software (Applied Spectral Imaging, Israel). These text files were loaded into RStudio as matrices. Relative filter settings were applied to remove unwanted signals, such as background (dark pixels). The low-wavelength matrix is used to filter out the high-intensity noise because high intensities in the low-wavelength matrix reflect bright noise. On the other hand, the high wavelength matrix is used to filter out the dark background noise. Filtering out the unwanted pixels should be done carefully with a robust reference interval to have reproducibility. Gaussian normal distribution of the pixels was a suitable reference interval for that (Figure S2). Calculating the mean value (μ) and using fixed values of standard deviation (σ) from the mean value enabled the upper cutoff limit and lower cutoff limit to be set to the low wavelength (500 nm) and high wavelength matrices (540 nm). The general guidelines describing how to set the cutoff limits for different genotypes based on the image outlook are found in Table S2. After applying the filter setting, the ratio between the low- and high-wavelength matrixes is calculated and stored in a matrix. This new calculated ratio matrix is further used to illustrate the data. A heatmap plot is generated to justify the effectiveness of the filter setting (Figure S3B–D). Moreover, the ratio matrix is used to generate a violin plot, with features similar to those of the ratiometric plot of ROI, but now on a larger full image scale (Figure S3E). Density plots were generated to visualize the ratio pixel distributions for images of each genotype or over the age of the same genotypes. Violin plots and pixel density distribution curves for the emission ratios for all images are found in Figures S3E, 4C–E, and S4.

### Fibril Formation of Recombinant Aβ Peptides, Hyperspectral Microscopy, and Transmission Electron Microscopy

In vitro fibril formation, hyperspectral microscopy, and transmission electron microscopy of recombinant Aβ1–40 and Aβ1–42 peptides were performed as described before.^31^ In short, the peptides were purchased from rPeptide, dissolved in 2 mM NaOH, and stored as stocks at −20 °C at a concentration of 1 mg/mL. At the time of fibrillation, the peptides were diluted to a concentration of 10 μM in PBS and fibrillated at 37 °C without shaking. For transmission electron microscopy (TEM), samples were collected at the beginning of the equilibrium phase for fibril formation, as deduced by ThT fluorescence. Carbon-coated copper grids were used to prepare TEM samples of fibrils negative stained with uranyl acetate. TEM images were collected by using a Jeol JEM 1230 microscope at 100 kV using a Gatan CCD camera. For hyperspectral microscopy, fibrils were collected at the end point (25 h). qFTAA and hFTAA were added to final concentrations of 13 and 7 nM respectively and left to sediment by gravity at room temperature overnight. Three microliter samples from the bottom of the tube were placed on superfrost + glass slides and dried in ambient air before mounting with Dako fluorescence mounting medium and coverslip. Images were collected as described for mouse tissue. Fluorescence spectra from ROIs covering the area of 2 representative images were collected, and the ratio between emission intensities at 500 and 540 nm was calculated.

## Supporting Information Available

Summary of Alzheimer’s disease mouse models used in the study; recommended values for filter setting for mouse tissue in unbiased image analysis in RStudio; fluorescence intensity ratiometric plot derived from the region of interest (ROI) within plaque core of *App*^*NL-F*^ and APP23 mice at different ages, measured at fluorescence intensity 500 and 540 nm; graphical representation of image filtration using relative filter setting in RStudio; and from hyperspectral images to pixel distribution ratiometric analysis in RStudio of APP23, APPPS1, and *App*^*NL-F*^ mice at intensities *I*_500_/*I*_540_ and *I*_500_/*I*_588_; and overview of pixel intensity distribution of *App*^*NL-F*^ mice at different age groups (PDF)

## Abbreviations

AβPP: Aβ precursor protein
AD: Alzheimer′s disease
CAA: cerebral amyloid angiopathy
fAD: familial Alzheimer′s disease
hFTAA: heptaformylthiopheneacetic acid
LCO: luminescent conjugated oligothiophene
PS1: presenilin 1
qFTAA: quatroformylthiopheneacetic acid
sAD: sporadic Alzheimers disease
