# Supplementary material for: Divergent Age-Dependent Conformational Rearrangement within Aβ Amyloid Deposits in APP23, APPPS1, and AppNL-F Mice
Source: ACS Chem Neurosci. 2024 Apr 23;15(10):2058–69. doi: 10.1021/acschemneuro.4c00104 (PMC11099915; doi:10.1021/acschemneuro.4c00104)
Supplement: Supplementary file 1 — cn4c00104_si_001.pdf [file cn4c00104_si_001.pdf]

Divergent age-dependent conformational rearrangement within Aβ amyloid deposits in APP23, APPPS1, and App^{NL-F} mice

Supporting information

Farjana Parvin[1], Samuel Haglund[1], Bettina Wegenast-Braun[2,3], Mathias Jucker[2,3], Takashi Saito[4,5], Takaomi C Saido[4], K Peter R Nilsson[1], Per Nilsson[6], Sofie Nyström[1*], Per Hammarström[1*]

[1] *Department of Physics, Chemistry and Biology (IFM), Linköping University, 58183 Linköping, Sweden*
[2] *German Center for Neurodegenerative Diseases (DZNE), University of Tübingen, 72076 Tübingen, Germany*
[3] *Hertie Institute for Clinical Brain Research, University of Tübingen, 72076 Tübingen, Germany*
[4] *Laboratory for Proteolytic Neuroscience, RIKEN Center for Brain Science, Wako, Saitama 351-0198, Japan*
[5] *Department of Neurocognitive Science, Nagoya City University Graduate School of Medical Sciences, Nagoya 467-8601, Aichi, Japan.*
[6] *Department of Neurobiology, Care Sciences and Society, Division of Neurogeriatrics, Karolinska Institutet, 17177 Solna, Sweden*

**corresponding authors*

Sofie Nyström, IFM-Chemistry Linköping University, 58183 Linköping, Sweden, +46739830893, sofie.nystrom@liu.se
Per Hammarström, IFM-Chemistry Linköping University, 58183 Linköping, Sweden, +46708141235, per.hammarstrom@liu.se

Supporting information contains:

Supporting tables

Table S1. Overview of the Alzheimer's disease mouse models used in the study.

Table S2. A guideline to choose the values for filter setting for whole image analysis of mouse tissue.

Supporting figures

Fig. S1. LCO fluorescence ratios I_{500}/I_{540} of different age groups of *App*$^{NL\text{-}F}$ and APP23 mice.

Fig. S2. Filtration settings of LCO fluorescence data using a relative filter setting in RStudio.

Fig. S3. Generation of filtered LCO data for pixel violin plots and density plots in RStudio.

Fig. S4. Pixel density distributions of qFTAA/hFTAA at different ages of *App*$^{NL\text{-}F}$ mice compared to APP23 and APPPS1 mice.

Supporting tables

Table S1. *Overview of the Alzheimer's disease mouse models used in the study*

**Mouse models/strains**	**APP23**	**APPPS1**	***App^{NL-F}***
Generation	First	First	Second
**Genetic modification**	**Transgenic**	**Transgenic**	**Knock-in (KI)**
**Promoter**	**mouse Thy1 element**	**mouse Thy1 element**	**mouse endogenous AβPP**
Humanized gene(s)	huAβPP^{751}(Swe)	huAβPP^{695}(Swe); huPSEN1(L166P)	huAβPP^{751}(Swe)
Mutations	AβPP $^{KM670/671NL}$	AβPP $^{KM670/671NL;}$ PS1^{L166P}	AβPP $^{KM670/671NL,I716F}$
**AβPP expression level**	**7-fold overexpression**	**3-fold overexpression**	**1-fold expression**
**Ratio of Aβ amyloid (Aβ42/Aβ40)**	**0.2-0.42***	**2.8-4.3***	**11-8600****
Availability	The Jackson Laboratory #stock 030504	Mathias Jucker & The Jackson Laboratory #stock 3765351	Takaomi Saido
Reference	Sturchler-Pierrat, C., et al., Proc Natl Acad Sci U S A, 1997. 94(24): p. 13287-92.	Radde, R., et al., EMBO Rep., 2006. 7(9): p. 940-6.	Saito, T., et al., Nat Neurosci, 2014. 17(5): p. 661-3.

* 6 - 25 months for APP23
6 - 18 months for APPPS1

* * 6 - 21 months for *App^{NL-F}*

Ye, L., et al., EMBO Rep., 2017.18(9): p. 1536-1544.

Heilbronner, G., et al., EMBO Rep., 2013.14(11): p. 1017-1022

Table S2: *A guideline to choose the values for filter setting for mouse tissue.*

Outlook of the image	Lower limit	Upper limit
When the background is completely dark, the tissue does not show any fluorescence. More yellow fluorescence appears in the image than blue fluorescence.	μ	infinity (μ+1000σ)
When the background is not completely dark but blurred with another color. The tissue shows intense blue fluorescence.	μ+1σ	infinity (μ+1000σ)

Supporting Figures S1-S4

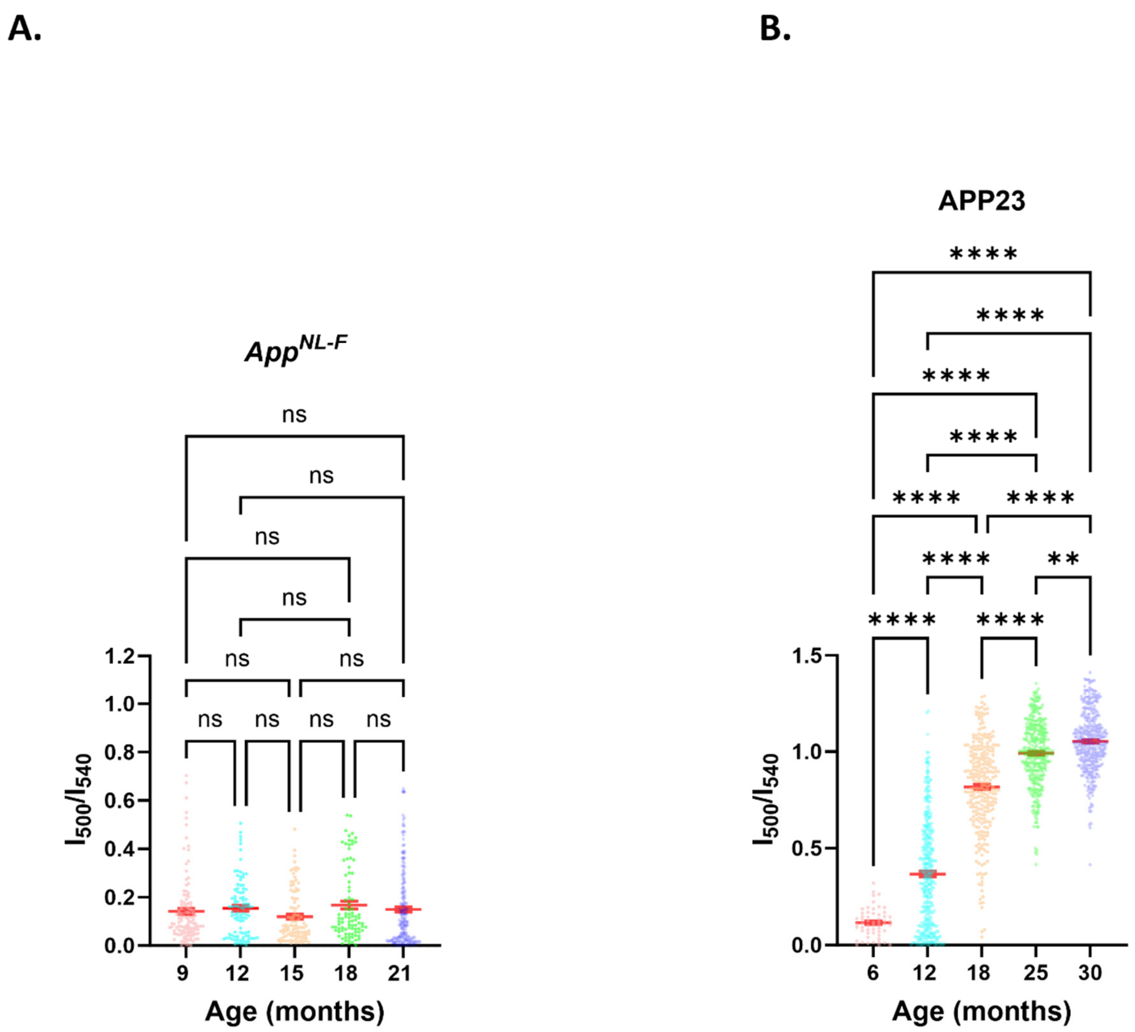

***Figure S1**: (A) Fluorescence intensity ratiometric plot from the region of interest (ROI) from plaque cores of App^{NL-F} mice at different age groups calculated at fluorescence intensity at 500 nm and 540 nm. Fluorescence intensity data from 3 mice at 9 months, 1 mouse at 12 months, 1 mouse at 15 months, 1 mouse at 18 months, and 3 mice at 21 months were analysed. The error bars represent SEM. An ordinary one-way ANOVA test was performed in GraphPad Prism for statistical analysis, ns = non-significant. The statistical analysis shows there is no significant change in fluorescence intensity ratio over time in this mouse genotype. (B) Fluorescence intensity ratiometric plot for APP23 mice at different age groups calculated from the region of interest (ROI) from plaque cores at fluorescence intensity at 500 nm and 540 nm. For the ratiometric plot, fluorescence intensity data from 5 mice at 6 months, 7 mice at 12 months, 5 mice at 18 months, 5 mice at 25 months and 5 mice at 30 months of age were analysed. An ordinary one-way ANOVA test shows significant differences in fluorescence intensity among different age groups, where ** = $p < 0.01$ and **** = $p < 0.0001$.*

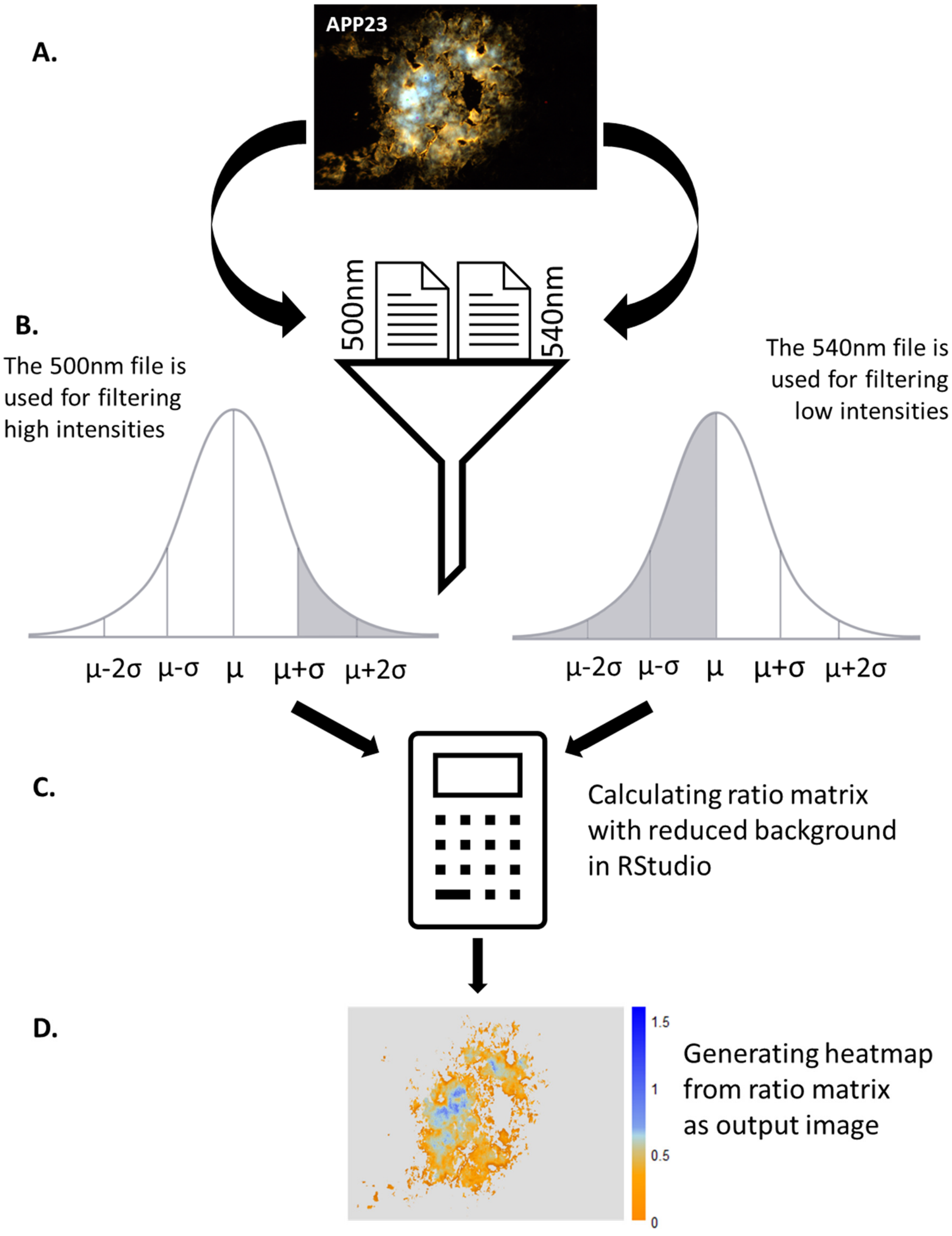

Figure S2: *A schematic overview of image filtration using a relative filter setting in RStudio. (A) Overview of the raw image. (B) Text files at different wavelengths i.e., at 500 nm, 540 nm and 588 nm is extracted from the hyperspectral microscope and used to apply relative filter setting to remove unwanted high and low intensities from the raw image. (C) Ratio matrix is calculated using the filtered text files at desired wavelengths i.e., at* I_{500}/I_{540}*nm or* I_{500}/I_{588}*nm in RStudio. (D) A heatmap is generated from the ratio matrix as an output image without unwanted signal.*

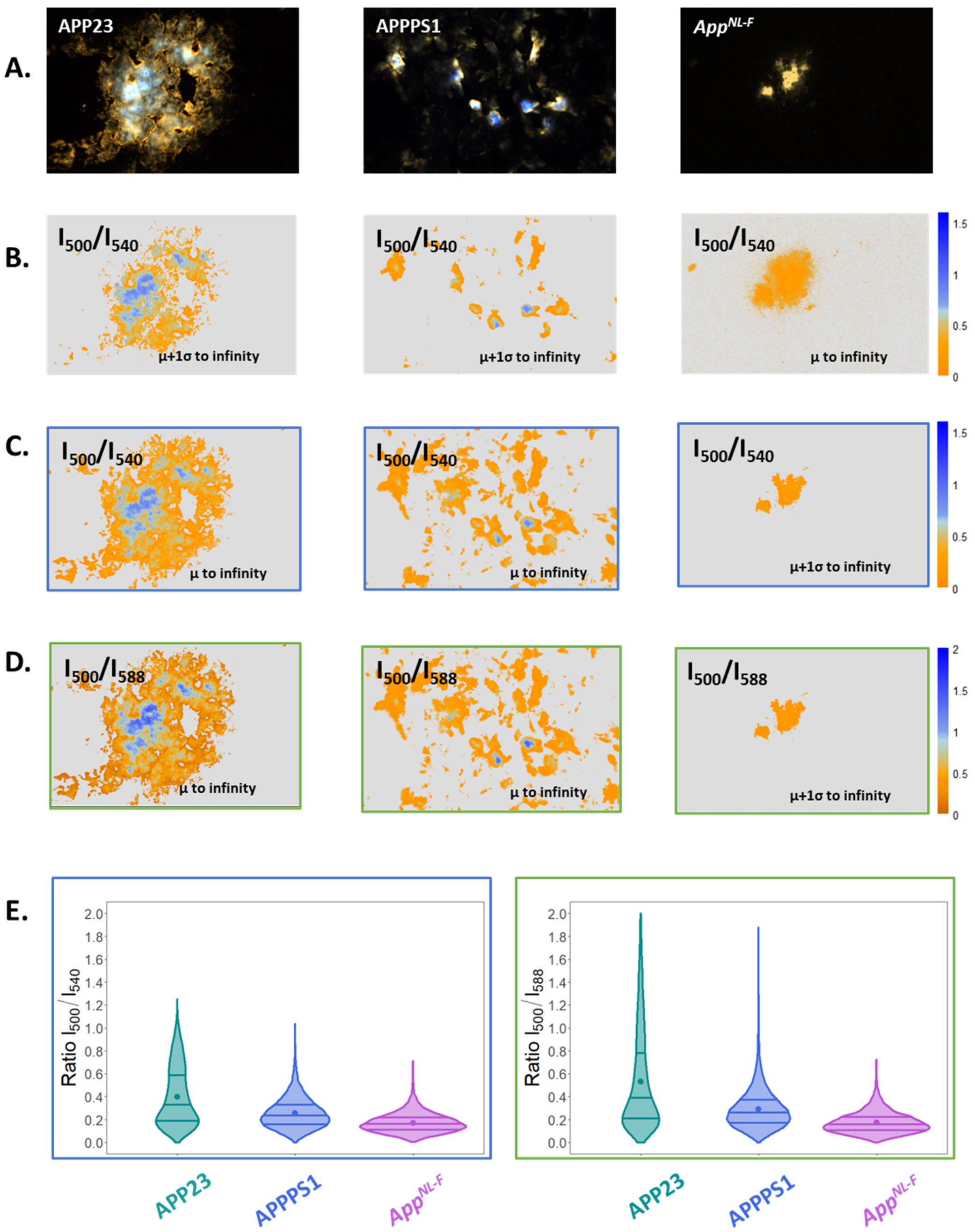

Figure S3: *(A) Overview of hyperspectral images of qFTAA and hFTAA stained amyloid plaques from APP23, APPPS1 and App$^{NL\text{-}F}$ mouse before applying the relative filter settings to the images. (B) Overview of the same plaques after applying the filter setting that is suboptimal for these genotypes i.e., APP23, APPPS1 and App$^{NL\text{-}F}$. (C) Overview of the plaques with the best relative filter setting at the intensity ratio of I_{500}/I_{540} nm encircled with the blue box. For APP23 and APPPS1 mice lower limit of filter setting is µ, meanwhile for App$^{NL\text{-}F}$ mouse is µ+1σ. The upper limit is infinity for all the mouse models. (D) Overview of the plaques with the best relative filter setting at the intensity ratio of I_{500}/I_{588} nm encircled with the green box. The filter setting for all the genotypes is the same as at the intensity ratio I_{500}/I_{540} nm. (E) The violin plots represent the pixel distribution ratiometric analysis of the different genotypes at intensities I_{500}/I_{540} nm and I_{500}/I_{588} nm, but herein considering all the pixels from images instead of selected ROIs. Each violin plot comprises 10 filtered images from each genotype. The dots in each violin plot represent the mean value of the corresponding genotype. The straight lines from the bottom of the violin plot represent the 1st quartile (25% data below this line), 2nd quartile or median (50% data below this line) and 3rd quartile (75% data below this line) respectively.*

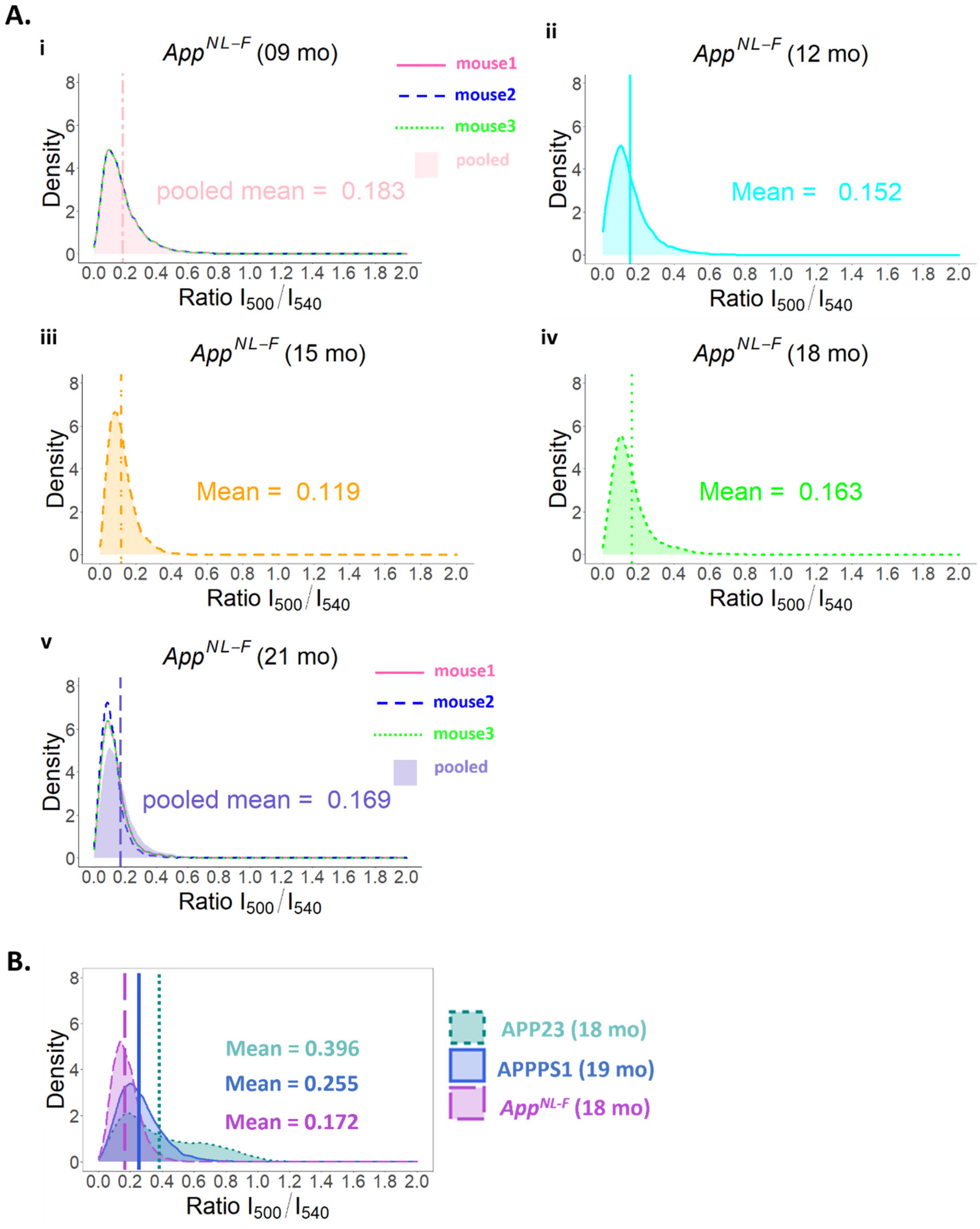

Figure S4*: (A) Individual pixel density distribution plots of App^{NL-F} mice at different age groups: i) at 9 months with a mean intensity ratio vale 0.183 pooled from 3 different mice ii) at 12 months with a mean intensity ratio value of 0.152 obtained from a single individual mouse iii) at 15 months with a mean intensity ratio vale 0.119 obtained from a single individual mouse iv) at 18 months with a mean intensity ratio vale 0.163 obtained from a single individual mouse and v) at 21 months with a mean intensity ratio vale 0.163 again pooled from 3 individual mice. (B) Overlay of pixel density distribution plots of APP23, APPPS1, and App^{NL-F} aged mice (at 18-19 months) compiled from 10 images from a single mouse from each genotype. APP23 mouse shows mean intensity ratio value of 0.396, APPPS1 mouse has mean intensity ratio value of 0.255 and App^{NL-F} mouse has mean intensity ratio value of 0.172.*
